# Pathogen-induced hijacking of host SUMOylation: from molecular mechanisms and prospects for therapeutic modulation

**DOI:** 10.3389/fimmu.2025.1686880

**Published:** 2025-12-01

**Authors:** Sandhya Padmakumar, Aravind Madhavan, Bipin G. Nair, Geetha B. Kumar

**Affiliations:** Amrita School of Biotechnology, Amrita Vishwa Vidyapeetham, Kollam, India

**Keywords:** post-translational modifications, SUMOylation, host-pathogen interaction, therapeutic strategies, small ubiquitin-like modifier (sumo) proteins

## Abstract

Post-translational modifications (PTMs) serve as essential regulatory mechanisms that fine-tune protein function, stability, localization, and interaction networks, enabling cells to adapt rapidly to physiological and pathological cues. Among the diverse PTMs, SUMOylation—the covalent attachment of Small Ubiquitin-like Modifier (SUMO) proteins to specific lysine residues on target substrates—has emerged as a dynamic and reversible modification with far-reaching implications in cellular homeostasis. Beyond its well-established roles in transcriptional regulation, DNA repair, and stress responses, recent studies highlight how pathogens have evolved to hijack the host SUMOylation machinery to subvert immune signalling, dampen inflammatory responses, and enhance intracellular survival. This review delves into the multifaceted role of SUMOylation in infectious disease, emphasizing its interplay with key host signalling cascades/axes such as NF-κB, MAPK, JAK-STAT, and interferon pathways. We explore how bacterial, viral, and fungal pathogens manipulate SUMOylation to reprogram host chromatin, modulate vesicular trafficking, and evade cytokine-mediated defences. Additionally, we examine the crosstalk between SUMOylation and other PTMs—such as ubiquitination, phosphorylation, and acetylation—that collectively shape the host-pathogen interface. By synthesizing current evidence on pathogen-driven SUMO modulation, we offer an integrated view of how this modification governs immune outcomes. Lastly, we evaluate emerging therapeutic strategies aimed at targeting SUMOylation pathways through small molecule inhibitors and genetic tools, with the goal of restoring immune competence and mitigating persistent infections. These insights position SUMOylation as a critical regulatory node and a promising target for host-directed therapies against infectious diseases.

## Introduction

The molecular interaction between pathogens and the host immune system is an intricate and dynamic process. While the host immune system deploys a multi-layered defence involving both innate and adaptive responses, pathogens have evolved and adapted several sophisticated strategies to bypass or subvert these defences (1). One of the most effective ways pathogens manipulate host biological systems is through interference with PTMs, which regulate protein function, stability, and cellular localization (2). Among these PTMs, SUMOylation—the covalent attachment of Small Ubiquitin-like Modifier (SUMO) proteins to lysine residues on substrates—has emerged as a key regulatory process governing host cellular signalling and immune response to infection (3).

SUMOylation is a highly dynamic and reversible PTM that modulates various cellular processes, including DNA repair, transcriptional regulation, protein stability and nuclear-cytoplasmic transport (4). The process is initiated by maturation of SUMO precursors by SUMO-specific proteases (SENPs), followed by activation via an E1 heterodimer (SAE1/SAE2), conjugation by the E2 enzyme UBC9, with substrate specificity conferred through E3 ligases (5). This enzymatic cascade culminates in the transfer of SUMO protein to target lysine residues on substrates that harbour specific SUMO consensus motifs (6). DeSUMOylation is regulated by a family of SENPs, ensuring timely removal of SUMO and restoring the original functional state of the target protein (7).

Humans express at least five SUMO isoforms—SUMO1, SUMO2, SUMO3, SUMO4 and SUMO5—each with distinct sequence identity, expression profiles and functional roles (8) SUMO1 primarily localizes to nuclear bodies, regulating chromatin organization and transcription repression. SUMO2 and SUMO3 are almost indistinguishable (~95% sequence identity) and act as stress-inducible regulators, mediating responses to genotoxic and oxidative stress by modifying transcription factors and DNA repair proteins (9). SUMO4, although tissue-specific and less characterized, is implicated in innate immunity and inflammatory signalling, while SUMO5, identified more recently, participates in promyelocytic leukaemia nuclear body (PML-NB) dynamics and polySUMOylation, particularly under infection-related stress (10). These paralogs demonstrate complex spatial and functional compartmentalization that orchestrates cell fate decisions and immune outcomes.

Pathogens from all domains—bacteria, viruses, and fungi—have developed specialized effectors that target and manipulate the host SUMOylation machinery, enabling them to promote their own survival. Pathogens such as *Salmonella enterica*, *Mycobacterium tuberculosis*, and *Legionella pneumophila* strategically manipulate host SUMOylation by various methods, either by suppressing it or by redirecting its targets to hinder autophagy, disrupt vesicular trafficking and reprogram NF-κB signalling, thereby creating conditions that support their survival and replication (11, 12). Similarly, viruses like herpes simplex virus (HSV-1), influenza A, Epstein-Barr virus (EBV), HIV and SARS-CoV-2, hijack the host SUMOylation machinery to dampen antiviral immunity (13). However, fungal pathogens such as *Candida albicans*, *Aspergillus fumigatus* and *Cryptococcus neoformans* use their own SUMOylation for survival, but its interaction with the host SUMOylation is just beginning to be understood (14).

Among traditional PTMs, SUMOylation is intricately interconnected with other PTMs such as ubiquitination, Neddylation, and ISGylation, often competing for modification sites, sequentially regulating substrate fate, or forming combinatorial modification patterns that collectively fine-tune protein stability, localization, and function (15). This crosstalk is exploited by pathogens to selectively stabilize or degrade host immune regulators. Viral and bacterial proteins may also harbour SUMO-interacting motifs (SIMs), enabling them to bind SUMOylated host proteins and alter their function or localization (16). Despite significant advances, the mechanisms by which SUMOylation is dynamically regulated in response to infection, particularly by fungal pathogens, are not fully elucidated. The interplay between SUMO and other PTMs in shaping immune responses remains a developing area of study. Understanding the spatiotemporal regulation of SUMOylation in different immune cells during infection will provide insights into new therapeutic targets. Furthermore, the potential for targeting SUMO-related enzymes or pathogen-SUMO interactions offers promising avenues for anti-infective strategies.

This review explores the role of SUMOylation across bacterial, viral and fungal infections, emphasizing its impact on immune signalling, chromatin remodelling and intracellular survival. We further discuss how SUMOylation interfaces with other host signals and evaluate its potential as a therapeutic target in infectious diseases. n this review, figures illustrate representative molecular interactions, highlighting how bacterial, viral, and fungal pathogens exploit host SUMOylation at key immune checkpoints, whereas tables summarize experimentally validated pathogen–SUMO interactions across species, providing a comparative overview of known host targets, effector proteins, and associated outcomes.

## SUMOylation in bacterial pathogenesis

**Figure 1:**
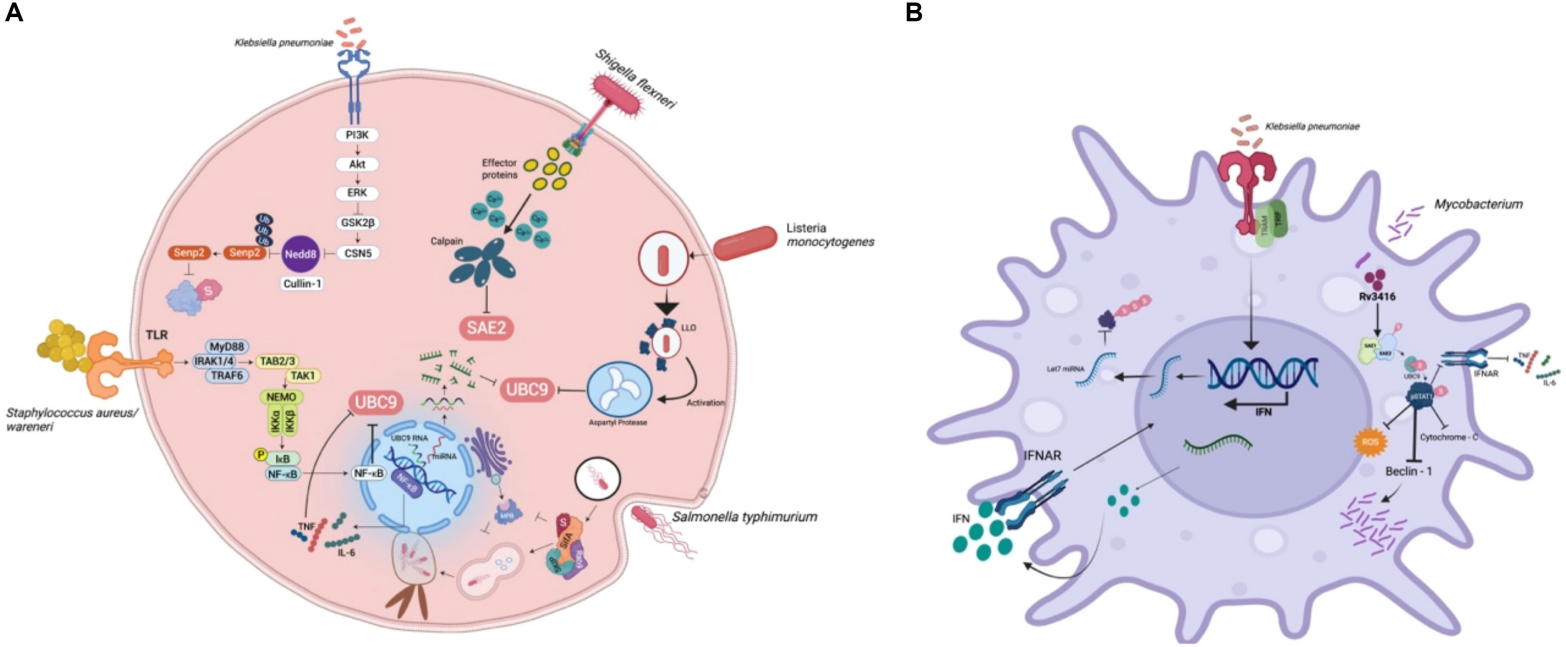
Bacterial modulation of host SUMOylation in epithelial and macrophage cells: **(A)** In epithelial cells, pathogens such as *Klebsiella pneumoniae*, *Listeria monocytogenes*, *Shigella flexneri*, and *Staphylococcus aureus/warneri* interfere with SUMOylation to suppress NF-κB activation and downstream cytokine production. **(B)** In macrophages, *Klebsiella pneumoniae* and *Mycobacterium tuberculosis* manipulate SUMOylation to dampen innate immune signalling. SUMO modification of pSTAT1 and IFNAR components suppresses ROS generation, autophagy, and cytokine output, while SUMO-linked microRNA regulation limits IFN responses. These events collectively reprogram macrophage function and favour bacterial persistence.

Bacterial pathogens have evolved sophisticated mechanisms that interfere with host immune signalling, often by exploiting the SUMOylation machinery, thereby establishing infection, altering inflammatory responses and creating favourable replication environments. SUMOylation of host proteins during bacterial infections alters the regulation of transcription factors, vesicle trafficking components and autophagy regulators, enabling bacteria to evade immune detection and persist intracellularly (17). Pathogens establish their pathogenicity by modulating host SUMOylation pathways within diverse host cell types, including immune and epithelial cells, thereby influencing processes such as inflammation, apoptosis, and intracellular persistence. For instance, the hypervirulent (hvkp) *Klebsiella pneumoniae* strain CIP52.145 has been shown to suppress the innate immune response in macrophages by broadly reducing global SUMOylation—particularly downregulating the let-7 miRNA, which in turn decreases SUMO1 levels. Uniquely, in epithelial cells, the hvkp*. pneumoniae* strain, exploits host SENP2 deSUMOylase stabilization via CSN5-mediated inhibition of Neddylation, strategically inducing reduction of global SUMOylation, to promote bacterial survival and immune evasion (18). In epithelial cells, bacterial effectors interfere with SUMOylation to block NF-κB activation (**Figure 1A**). In macrophages, *Klebsiella pneumoniae* and *Legionella pneumophila* alter the SUMO-E2/E3 conjugation enzymes to impair phagosome maturation and intracellular killing (**Figure 1B**). The bacterium also downregulates type I interferon responses by impairing SUMO-dependent modification of STAT proteins. These modifications allow *K. pneumoniae* to persist within macrophages by limiting inflammatory feedback and promoting an immunosuppressive environment (18, 19). *Mycobacterium tuberculosis* (Mtb) manipulates host SUMOylation in a coordinated manner to subvert immune defences and promote intracellular survival. Upon infection, Mtb triggers oxidative stress, which alters the SUMOylation status of key transcription factors, leading to reduced macrophage activation. Concurrently, Mtb induces deSUMOylation of STAT1 and chromatin-associated transcription factors, dampening interferon-γ–mediated signalling and reshaping histone architecture to repress immune gene expression. In parallel, Mtb targets SUMO-dependent vesicle trafficking machinery, disrupting autophagy initiation and arresting phagosome maturation. This multifaceted interference—spanning transcriptional silencing, signal transduction suppression and vesicular pathway blockade—forms an integrated strategy that enables Mtb to evade immune clearance and persist within host macrophages (20). While pathogens like *Klebsiella pneumoniae* and *Mycobacterium tuberculosis* broadly interfere with phagosome maturation, *Salmonella* Typhimurium (S. Tm) strain SL1344 and *Legionella pneumophila*, specifically modulate the SUMOylation of selected host proteins involved in vesicle trafficking. Intracellular pathogens such as *Salmonella enterica* serovar Typhimurium (SL1344) and *Legionella pneumophila* have evolved sophisticated strategies to manipulate the host SUMOylation landscape, thereby remodelling vesicular trafficking pathways to support niche formation (21, 22). *S. Typhimurium* delivers a suite of Type III secretion system (T3SS) effectors that perturb SUMO conjugation dynamics on SNARE proteins and autophagy-associated regulators. This targeted modulation hinders autophagosome maturation and its fusion with lysosomes, culminating in the generation of the *Salmonella*-containing vacuole (SCV), a membrane-bound compartment that supports intracellular replication, while evading degradative pathways (21). In parallel, *L. pneumophila* hijacks SUMO-modified Rab GTPases and endosomal SNAREs to engineer the Legionella-containing vacuole (LCV). Additional effectors further impede lysosomal fusion, thus preserving the stability of the LCV. Collectively, these pathogens exemplify a convergent evolutionary tactic, wherein modulation of SUMOylated trafficking components subverts host endo-lysosomal integrity to favour vacuole persistence and immune evasion (22). Several mucosal and cytosolic bacterial pathogens have evolved mechanisms to manipulate host SUMOylation pathways to facilitate immune evasion, inflammation and persistence. *Listeria monocytogenes*, a cytosolic pathogen, disrupts host SUMOylation during early stages of infection through its pore-forming toxin, listeriolysin O, which impairs SUMO-conjugating enzymes and promotes proteasomal degradation of UBC9, the central E2 ligase. This leads to a global reduction in SUMOylation of immune signalling molecules, allowing Listeria to evade type I interferon responses and enabling actin-based intracellular motility and cytosolic dissemination (23). In contrast, *Helicobacter pylori*, a gastric mucosal pathogen, associated with chronic inflammation and cancer, uses its virulence factor CagA, to enter the host nucleus and interact with SUMO-modified transcriptional regulators such as p53, NF-κB and β-catenin (24). These interactions disrupt normal control of epithelial proliferation, inflammation, and apoptosis, thereby contributing to chronic infection and gastric oncogenesis. Similarly, *Staphylococcus warneri*, a coagulase-negative commensal with emerging pathogenic potential, targets epithelial SUMOylation during intestinal colonization. The bacterium secretes the pore-forming toxin warnericin RK, which reduces SUMO conjugate levels in epithelial cells, particularly impacting transcription factors like STAT3 and NF-κB. This disruption compromises epithelial barrier function and suppresses antimicrobial peptide production, leading to increased intestinal inflammation and thereby creating a niche favourable for colonization and opportunistic infection (25). Pathogen-induced SUMO remodelling not only reprograms host transcription but also correlates with increased bacterial load and dampened cytokine secretion, thereby influencing disease progression and host survival (26). In bacterial infections, such modulation has been associated with reduced production of IL-6, TNF-α, and IFN-γ, compromising macrophage activation and pathogen clearance (27). Collectively, these pathogens as in **Table 1** exemplify distinct strategies, by which interference with SUMOylation at transcriptional and epithelial regulatory nodes, contributes to immune modulation and host-pathogen adaptation. These examples also illustrate how diverse bacterial pathogens subvert host SUMOylation at multiple levels—ranging from global SUMO suppression, to specific targeting of immune signalling nodes. The role of SUMOylation in regulating vesicular trafficking, transcriptional activation and cytokine release makes it a crucial axis for bacterial immune evasion and it is represented in epithelial cells and macrophages separately in **Figures 1A, B** respectively.

**Table 1:** Bacterial modulation of host SUMOylation: effectors, targets, and mechanisms.

Bacterium	Bacterial effectors	Target proteins	SUMOylation proteins	SUMO sites	Mechanism	Disease	References
*Klebsiella pneumoniae*	Uncharacterized effector(s)	SUMO-conjugating enzymes (UBC9), IκBα, STAT1/2	SUMO1, SUMO 2/3	Lysine residues on UBC9 and immune regulators	Reduces global SUMOylation to suppress immune responses	Pneumonia, sepsis, UTIs	(18)
*Staphylococcus warneri*	Warnericin RK	STAT3, NF-κB components	SUMO2/3	STAT and NF-κB associated SUMO sites	DeSUMOylation of epithelial transcription factors to promote inflammation	Gut inflammation, opportunistic infections	(25)
*Mycobacterium tuberculosis*	ESAT-6, others	Histone modifiers, STAT1, chromatin regulators	SUMO1, SUMO2	Histone tail lysine’s, IRF-linked sites	Alters SUMOylation to suppress IFN-γ signalling and promote latency	Tuberculosis	(20)
*Legionella pneumophila*	LubX, SidE family	Rab7, SUMO E3 ligase targets	SUMO1, SUMO2	Rab7 and ER-trafficking SUMO motifs	Hijacks SUMOylated Rab7 to arrest phagosome maturation	Legionnaires’ disease	(22)
*Listeria monocytogenes*	Listeriolysin O, Internalins	UBC9, IRFs	SUMO1	UBC9 and transcription factors	Degrades UBC9, impairing global SUMOylation and innate responses	Listeriosis	(28)
*Salmonella enterica*	SPI-2 effectors	SNARE regulators, vesicle trafficking proteins	SUMO2/3	Lysine’s near SNARE recognition domains	Modulates SUMOylation of trafficking proteins to remodel vacuoles	Typhoid fever, gastroenteritis	(29)
*Helicobacter pylori*	CagA	NF-κB subunits, p53	SUMO1	SUMO target motifs in p53 and NF-κB	CagA interferes with SUMO-modified signalling pathways	Gastric ulcers, gastric cancer	(30)
*Shigella flexneri*	OspF, IpaH	Host chromatin remodels, MAPKs, NF-κB	SUMO1, SUMO2	Chromatin-associated lysine’s, phospho-SUMO motifs	Effector OspF interferes with chromatin SUMOylation to suppress inflammation	Shigellosis (dysentery)	(31)
*Escherichia coli (EHEC)*	EspF, NleB	TRAFs, death domain-containing proteins	SUMO1, SUMO2/3	NF-κB signalling-related SUMO motifs	NleB interferes with SUMO-modified apoptosis regulators to prevent host cell death	Haemorrhagic colitis, HUS	(32)
*Brucella abortus*	VirB effectors	Histone modifiers, immune signalling intermediates	SUMO2/3	Histone tail SUMO sites, cytokine promoter regions	Manipulates SUMOylation of chromatin enzymes to inhibit pro-inflammatory gene expression	Brucellosis	(33)

## SUMOylation in viral pathogenesis

Viruses are obligate intracellular pathogens that rely heavily on hijacking host cellular machinery for their replication, persistence and immune evasion. One of the key pathways exploited by viruses is host SUMOylation, a reversible post-translational modification that affects protein localization, stability, transcriptional activity and protein-protein interactions. Viruses can manipulate crucial host processes such as chromatin remodelling, immune signalling, DNA repair and stress responses, by modulating the SUMOylation landscape, thereby creating an intracellular environment beneficial to viral replication and immune escape (34).

**Figure 2:**
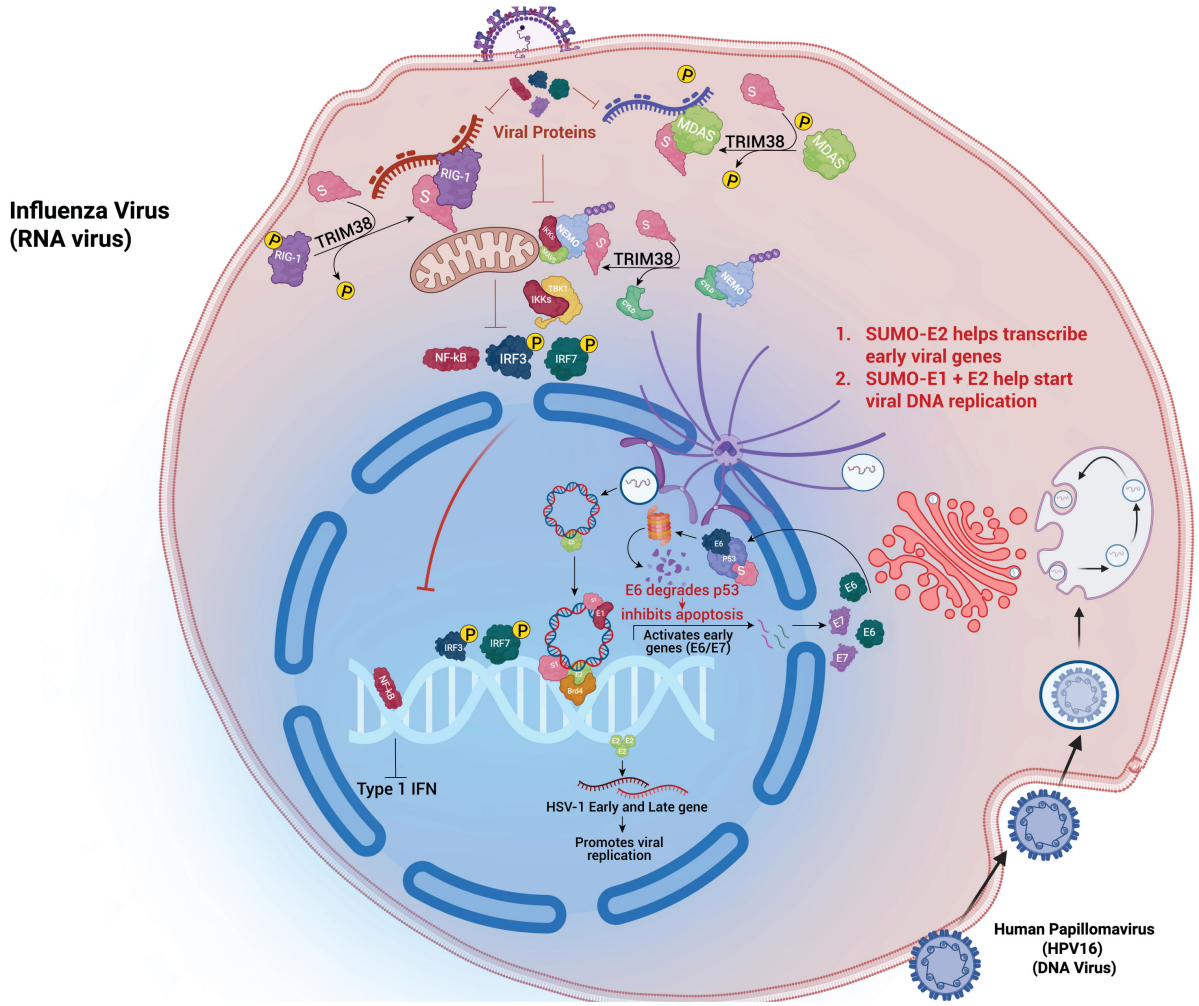
Viral hijacking of the host SUMO pathway: RNA and DNA viruses exploit host SUMOylation to regulate replication and immune evasion. *Influenza A virus* uses SUMO-E2 to enhance early gene transcription, while TRIM38-mediated SUMOylation of RIG-I and MDA5 modulates IFN responses. DNA viruses such as *HPV16* and *HSV-1* recruit SUMO enzymes to control p53 degradation and suppress IRF3/NF-κB activation, facilitating viral gene expression and replication.

An emerging theme in viral pathogenesis, as illustrated in **Figure 2**, is the SUMOylation of innate immune sensors—particularly cGAS and STING—which regulates antiviral signalling (35). TRIM38-mediated SUMOylation of cGAS and STING during early infection, stabilizes these proteins and enhances their signalling, promoting type I interferon production. Later, viral infections often induce deSUMOylation via host proteases like SENP2, targeting cGAS and STING for ubiquitin-mediated degradation, thereby shutting down interferon responses (36). SUMO modification of antiviral RNA-binding proteins, including members of the DDX and hnRNP families, also regulates RNA sensing and processing, influencing RIG-I–like receptor signalling and viral RNA recognition (37). These SUMO-dependent checkpoints are prime targets for viral manipulation, enabling a temporal “switch” from an early pro-immune to a later immunosuppressive state.

Viruses from diverse families have evolved mechanisms to either enhance or inhibit SUMOylation of specific host factors or viral proteins. This manipulation is often tightly linked to crosstalk with other host signalling pathways, particularly ubiquitylation, phosphorylation, interferon signalling and epigenetic regulation, allowing viruses to orchestrate multi-layered control over host defences. For instance, Herpes Simplex Virus-1 (HSV-1) utilizes its immediate-early protein ICP0 as a SUMO-targeted ubiquitin ligase (STUbL), to degrade the SUMOylated components of nuclear bodies like PML and Sp100. This action simultaneously involves ubiquitin-SUMO crosstalk, leading to the dismantling of antiviral nuclear structures and suppression of intrinsic immunity during reactivation (38, 39).

Kaposi’s Sarcoma-Associated Herpesvirus (KSHV), also exemplifies this interplay. Its SUMOylated LANA protein tethers the viral genome to host chromatin and recruits HDACs and polycomb repressive complexes (PRCs), coordinating SUMOylation with histone deacetylation and methylation to silence host immune genes during latency (40). Upon reactivation, KSHV actively disrupts SUMO-modified antiviral complexes, liberating transcription factors and facilitating lytic gene expression. This dynamic modulation reflects a coordinated SUMO–epigenetic–transcriptional axis.

In HIV-1, SUMOylation contributes to multiple stages of the viral life cycle. HIV integrase undergoes SUMO modification, enhancing its nuclear import and catalytic function, crucial for viral genome integration (41). In parallel, HIV modulates the SUMOylation status of host restriction factors like TRIM5α, reducing their antiviral activity (42). Furthermore, HIV exploits SUMOylation to control host immune regulators such as NF-κB and STAT1/3, where SUMOylation either represses or stabilizes these factors to maintain latency and evade immune surveillance (43). Notably, SUMOylation of IκBα or its upstream regulators can inhibit NF-κB activation, representing a direct SUMO–NF-κB signalling node manipulated by multiple viruses (44).

Influenza A virus deploys its NS1 protein to promote the SUMOylation of IRF3, repressing its transcriptional activity and consequently suppressing type I interferon (IFN-I) production (45). NS1 itself is SUMOylated, which enhances its interaction with host factors involved in immune modulation (46). This underscores a recurring theme in viral infections: feedback crosstalk between SUMOylated viral proteins and modified host immune effectors. Similarly, SARS-CoV-2 has been shown to interfere with SUMOylation of STAT1 and IRF3, leading to their cytoplasmic retention or degradation, thereby blunting IFN responses—a hallmark of severe COVID-19 (47).

Adenoviruses also exploit SUMOylation as a bridge to modulate the DNA damage response (DDR). The viral E1B-55K protein interacts with SUMO-conjugating enzymes to alter the SUMOylation of DNA repair proteins such as Mre11 and NBS1, disrupting the MRN complex and preventing ATM activation (48). This dampens apoptosis and promotes viral genome replication, linking SUMOylation to DDR inhibition and cell survival.

The Epstein-Barr Virus (EBV) employs EBNA1, which interacts with SUMOylated chromatin modifiers, facilitating the maintenance of viral episomes and latency while repressing antiviral genes (48). Importantly, EBV also modulates SUMOylation of DNA methyltransferases (DNMTs) and transcriptional repressors, highlighting a SUMO–epigenetic crosstalk that contributes to immune evasion and oncogenesis (49). Similarly, in viral infections, SUMO-mediated suppression of IRF3, STAT1, and NF-κB signalling attenuates interferon responses, leading to higher viral replication rates and severe disease phenotypes (43, 50).

Together, these examples as in **Table 2** underscore the fact that viral manipulation of the host SUMOylation pathway is not isolated—it is deeply entwined with other signalling networks, including ubiquitin-proteasome degradation, phosphorylation cascades, histone modification systems and cytokine responses. By leveraging these intersections, viruses fine-tune the host intracellular environment to either establish latency or maximize replication. Understanding these multi-layered interactions not only clarifies fundamental aspects of viral pathogenesis but also highlights the SUMO pathway as a potential therapeutic target—where inhibition could restore antiviral immunity and disrupt critical virus-host protein interactions.

**Table 2:** Viral hijacking of host SUMOylation machinery: strategies and consequences.

Virus	Viral proteins	Target proteins	SUMOylation proteins	SUMO sites	Mechanism	Disease	References
Herpes Simplex Virus (HSV-1)	ICP0	PML, Sp100	SUMO1, SUMO2/3	Various on PML and nuclear body components	ICP0 acts as STUbL, targeting SUMOylated proteins for degradation	Herpes simplex infections (encephalitis, cold sores)	(40)
Human Immunodeficiency Virus (HIV)	Integrase (IN)	TRIM5α, host transcription factors	SUMO1	Lysine residues on IN	SUMOylation enhances stability and nuclear import of IN; modifies host factors to suppress restriction	AIDS	(43)
Influenza A Virus (IAV)	NS1	IRF3, STAT1	SUMO1, SUMO2/3	Lysine residues on IRF3	NS1 modulates SUMOylation of IRFs to suppress interferon signalling	Influenza (respiratory illness)	(45)
Kaposi’s Sarcoma-associated Herpesvirus (KSHV)	LANA	Histone-modifying enzymes, chromatin anchors	SUMO2/3	SIM-containing motifs on chromatin regulators	LANA SUMOylation enables viral latency and host gene repression	Kaposi’s Sarcoma	(51)
Adenovirus	E1B-55K	p53, Mre11, NBS1	SUMO1	Lysine residues on p53-related proteins	E1B-55K interaction with SUMOylated proteins suppresses DNA damage response	Respiratory infections, tumor models	(52)
Epstein-Barr Virus (EBV)	BZLF1	PML, HDACs	SUMO1, SUMO2/3	SIM-containing regions on PML, HDACs	BZLF1 disrupts SUMOylated repressor complexes to reactivate EBV from latency	Infectious mononucleosis, cancers	(53)
Human Cytomegalovirus (HCMV)	IE1	STAT2, PML	SUMO1, SUMO2	Lysine residues on STAT2 and nuclear proteins	IE1 interferes with SUMOylation of STAT2 and PML to suppress innate immunity	Congenital CMV, retinitis, pneumonia	(54)
Hepatitis B Virus (HBV)	HBx	p53, chromatin modifiers	SUMO1	Lysine’s on transcriptional regulators	HBx modifies SUMOylation of chromatin remodels to enhance viral transcription	Hepatitis B, liver cancer	(50)
Zika Virus	NS5	STAT2, host transcription factors	SUMO1	SUMO sites near STAT2 domains	NS5 manipulates SUMOylated STAT2 to suppress type I IFN signalling	Zika fever, congenital defects	(55)
Ebola Virus	VP35	IRF3, TBK1	SUMO2/3	IRF-associated SUMO motifs	VP35 inhibits SUMO-modified IRF3/TBK1 to block interferon production	Ebola virus disease	(56)
Human T-cell Leukaemia Virus (HTLV-1)	Tax	NF-κB, CREB-binding protein	SUMO1, SUMO2/3	Tax-associated motifs on host transcription regulators	Tax modifies SUMOylation to deregulate transcription and promote T-cell transformation	Adult T-cell leukaemia/lymphoma	(57)
Hepatitis C Virus (HCV)	Core, NS5A	Host lipid regulators, IFN signalling proteins	SUMO1, SUMO2	SUMO motifs on IFN pathway members	SUMOylation changes aid in viral persistence and immune modulation	Chronic hepatitis C, liver cancer	(58)
Human Papillomavirus (HPV16)	E6, E7	p53, Rb	SUMO1	Lysine residues on tumor suppressor proteins	E6/E7 target SUMOylated tumor suppressors to promote cell cycle dysregulation	Cervical cancer, genital warts	(59)
SARS-CoV-2	Nsp3, ORF6	STAT1, IRF3	SUMO2/3	IRF/STAT SUMO consensus sites	Nsp3/ORF6 interfere with SUMO-modified IFN pathway to suppress host defence	COVID-19	(60)

## SUMOylation in fungal pathogenesis

**Figure 3:**
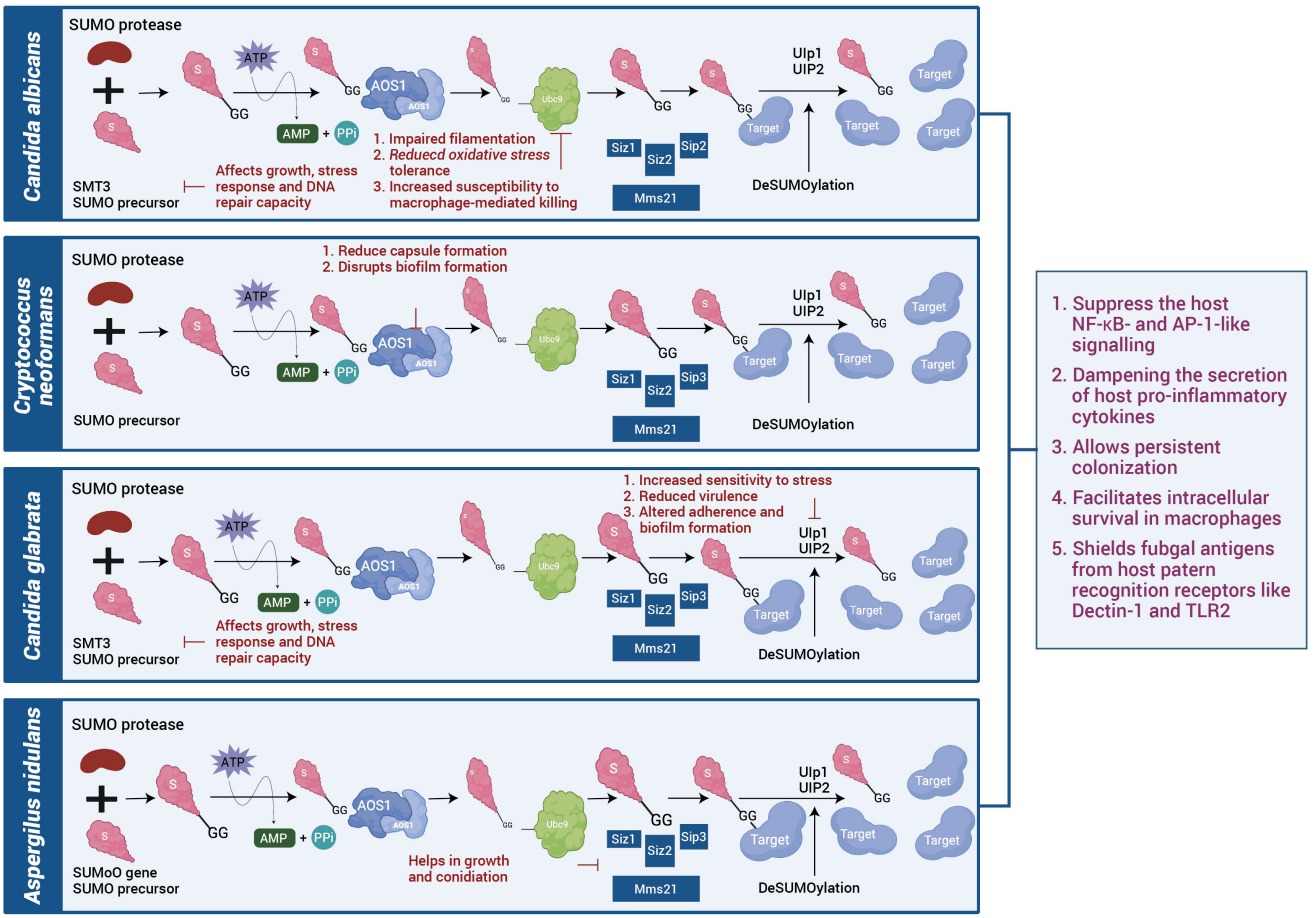
SUMOylation systems in fungal pathogens: Fungal species such as *Candida albicans*, *Cryptococcus neoformans*, *Candida glabrata*, and *Aspergillus nidulans* possess conserved SUMO conjugation and deSUMOylation machinery. Disruption of SUMO enzymes impairs stress tolerance, capsule or biofilm formation, and virulence. Comparative analysis suggests that SUMO signalling supports fungal adaptation and evasion of host immune defences.

Fungal pathogens employ their intrinsic SUMOylation machinery as a versatile regulatory system to survive hostile host environments, evade immune defences and maintain virulence. Unlike bacteria and viruses that actively manipulate host SUMOylation, fungi predominantly rely on their own SUMO-conjugation networks as shown in **Figure 3** to control critical cellular processes during infection (61). In *Candida albicans*, its own fungal SUMOylation modulates the yeast-to-hyphae transition, a morphological shift essential for tissue invasion and immune evasion. Loss of its SUMO pathway components such as UBC9 or SMT3 in *Candida albicans* results in impaired filamentation, reduced oxidative stress tolerance and increased susceptibility to macrophage-mediated killing (62). *Candida* also uses the fungal SUMOylation system to suppress the host NF-κB and AP-1-like signalling, dampening the secretion of host pro-inflammatory cytokines and allowing persistent colonization (63). Similarly, *Cryptococcus neoformans* exploits its SUMOylation machinery to modulate chromatin accessibility, driving the transcriptional repression of immune-related genes and enabling the pathogen to survive the acidic, oxidative, and nutrient-limited environment within macrophages. Disruption of SUMO-modifying enzymes in *Cryptococcus* impairs capsule biosynthesis, melanin production and overall virulence, underlining its dependence on SUMO signalling for pathogenesis (64). In *Aspergillus fumigatus*, SUMOylation coordinates the function of stress-responsive transcription factors governing MAPK and HOG signalling pathways, which are vital for thermotolerance and oxidative stress resistance (65). Fungal mutants lacking SUMO E2 enzymes such as AfUBC9 show compromised growth at body temperature, hypersensitivity to reactive oxygen species and attenuated infectivity *in vivo* (66). Thermally dimorphic fungi such as *Histoplasma capsulatum* and *Paracoccidioides brasiliensis* exploit SUMOylation to regulate phase transition between mycelial and yeast forms—a switch that facilitates intracellular survival in macrophages and shields fungal antigens from host pattern recognition receptors like Dectin-1 and TLR2 (67). Moreover, *Talaromyces marneffei*, an emerging pathogen in immunocompromised individuals, utilizes SUMOylation to modulate its temperature-dependent dimorphic switch and cell wall remodelling, which collectively contribute to its stealth and survival in host phagocytes. Beyond morphogenesis and stress adaptation, SUMOylation in fungi intersects with drug resistance mechanisms. In *Candida*, SUMO-regulated transcription factors influence the expression of efflux pumps and ergosterol biosynthetic enzymes, thereby enhancing azole resistance (68). In *Cryptococcus*, SUMO pathway components fine tune melanin synthesis and cell wall-associated proteins that confer resistance to amphotericin B and oxidative damage. Some filamentous fungi like *Fusarium oxysporum* and *Botrytis cinerea*, also rely on SUMOylation for pathogenicity-related processes, including toxin production, host tissue invasion and adaptation to oxidative environments, although these mechanisms are still under active investigation (69). Although direct evidence in mammalian pathogenic fungi is limited, plant pathogens such as Fusarium oxysporum and Botrytis cinerea exploit plant SUMO machinery to suppress defence responses, hinting at a conserved strategy. The parallels between plant and mammalian infection models suggest that fungal effectors may have evolved SUMO-targeting motifs to reprogram host stress and immune signalling pathways. While fungi have not yet been shown to directly alter host SUMOylation, their influence on cytokine signalling and host stress responses during chronic infection may secondarily modulate host SUMO pathways. Together, as in **Table 3** highlight that fungal SUMOylation functions as a master regulator of pathogenesis, stress resistance, morphogenesis and antifungal adaptation, making it a compelling target for therapeutic intervention in the era of rising fungal drug resistance.

**Table 3:** Fungal SUMOylation and host interaction: molecular components and pathogenic outcomes.

Fungus	Fungal effectors/mechanisms	Host target proteins	SUMOylation proteins	SUMO sites	Mechanism	Disease	References
Candida albicans	Stress-induced SUMOylation modulation	NF-κB, AP-1, stress-response factors	SUMO1, SUMO2/3	Lysine’s on transcription factors and chromatin proteins	Alters host transcription factor SUMOylation to suppress inflammation	Candidiasis (oral, systemic)	(68)
Aspergillus fumigatus	Oxidative stress-triggered SUMO remodelling	IRFs, cytokine transcription factors	SUMO1, SUMO2	Stress response-associated SUMO sites	Reduces SUMOylation of immune signalling proteins to evade detection	Invasive aspergillosis	(65)
Cryptococcus neoformans	Chromatin silencing via SUMO-modified regulators	Histone deacetylases (HDACs), chromatin remodels	SUMO1, SUMO2/3	Histone tail lysine’s, promoter-bound proteins	Induces SUMOylation of chromatin repressors to silence antifungal genes	Cryptococcal meningitis	(69)
Histoplasma capsulatum	Suppression of antigen presentation through epigenetic SUMO changes	MHC presentation regulators, histone modifiers	SUMO2/3	Epigenetic regulator-associated motifs	Modifies chromatin states via SUMO to reduce antigen presentation	Pulmonary and disseminated histoplasmosis	(67)
Paracoccidioides brasiliensis	Modulation of SUMOylation under thermal stress and host-like conditions	Stress granule components, HSF1	SUMO1, SUMO2	Thermo-responsive SUMO sites on chaperones and regulators	SUMOylation regulates thermotolerance and morphogenesis during infection	Paracoccidioidomycosis	(67)
Candida glabrata	Evades macrophage response via SUMO-dependent stress adaptation	Heat shock factors, stress-response elements	SUMO1, SUMO2	Stress-related transcription factor SUMO motifs	SUMO-mediated stress resistance enhances survival in immune cells	Candidiasis (systemic, resistant forms)	(70)
Aspergillus nidulans	Genetically characterized SUMOylation pathway involved in virulence	Histone-associated proteins, signalling enzymes	SUMO1, SUMO2/3	Histone tails, kinase substrates	Genetic SUMO disruption reduces virulence and stress resistance	Opportunistic aspergillosis in immunocompromised hosts	(65)

## SUMOylation crosstalk with other post-translational modifications in host–pathogen interactions

SUMOylation operates within a complex network of PTMs, including ubiquitination, phosphorylation, acetylation and Neddylation forming an integrated system that modulates host signalling, chromatin remodelling and immune responses (75. 78). Pathogens exploit these PTM intersections to hijack host processes for survival. SUMO–ubiquitin crosstalk is notably manipulated by viruses and bacteria. While SUMOylation may shield proteins from ubiquitin-mediated degradation, SUMO-targeted ubiquitin ligases (STUbLs) like ICP0 of Herpes Simplex Virus type 1 degrade SUMOylated nuclear restriction factors (71). Conversely, HIV-1 benefits from SUMOylated integrase and cofactors to enhance persistence (72). *Legionella pneumophila* employs LubX, a STUbL-like effector, to degrade SUMOylated host restriction proteins (73). Phosphorylation–SUMOylation crosstalk via phospho-dependent SUMO motifs (PDSMs) regulates SUMO conjugation (74). *Influenza A* NS1 protein phosphorylates IRF3 to enhance its SUMOylation and suppress IFN signalling, while *Salmonella* manipulates MAPK/JNK signalling to alter phosphorylation and indirectly modulate SUMOylation of immune regulators. Transcription factors such as p53 or NF-κB undergo SUMO–ubiquitin crosstalk during infection—these pathogen-triggered modifications differ from basal homeostatic SUMO control (75, 76). Neddylation and SUMOylation antagonistically regulate Cullin-RING ligases (CRLs). Viral agents like KSHV and EBV hijack this axis to alter host protein degradation (77). ISGylation, overlapping with SUMO targets, modulates antiviral responses via IRFs and STATs. Viruses like SARS-CoV-2 and *Influenza A*, disrupt this balance to suppress ISG15-mediated antiviral gene expression (78). Together, these PTM networks represent key regulatory hubs that pathogens selectively reprogram. Targeting SUMO–PTM crosstalk may offer a focused therapeutic strategy to counter infections without widespread immune activation.

## SUMOylation-mediated intracellular niche formation and epigenetic reprogramming

Intracellular pathogens have evolved to exploit host SUMOylation pathways to support two critical survival strategies: the formation of protected intracellular niches and the silencing of immune-responsive genes. For niche formation, pathogens like *Legionella pneumophila* secrete effectors such as LubX, that mimic STUbLs, leading to the degradation of SUMOylated vesicle trafficking proteins like Rab7. This interferes with endosome maturation and blocks lysosomal fusion, stabilizing the *Legionella*-containing vacuole (73). Similarly, *Mycobacterium tuberculosis* manipulates SUMOylation of syntaxins and Rab GTPases, while also activating SENPs to deSUMOylate host regulators, thus arresting phagosome maturation and maintaining survival within immature vesicles (79). *Salmonella enterica* targets SUMOylation of SNARE proteins, including VAMPs and syntaxins, to inhibit vacuole-lysosome fusion and remodel the *Salmonella*-containing vacuole (80).

In parallel, these pathogens use SUMOylation to reprogram host gene expression through epigenetic remodelling. SUMO-modified chromatin-associated enzymes such as histone deacetylases (HDACs), histone methyltransferases (e.g., SUV39H1), and chromatin remodelling complexes like SWI/SNF and NuRD suppress the transcription of cytokines, interferon-stimulated genes (ISGs), and antigen presentation machinery (81). *M. tuberculosis* enhances SUMOylation of SET-domain repressors and HDACs to silence nitric oxide and inflammatory gene expression, while also dynamically regulating this repression by activating SENPs (82). In fungal pathogens like Cryptococcus neoformans, SUMOylation of host chromatin remodels dampens immune gene expression, aiding in immune evasion and intracellular persistence (83).

Together, these findings highlight SUMOylation as a central regulatory node that pathogens exploit to modify both cellular architecture and gene expression, ensuring successful intracellular colonization and long-term survival.

## Therapeutic targeting of SUMOylation: insights and future perspectives

Targeting the SUMOylation pathway presents an emerging strategy for therapeutic intervention in infectious diseases, particularly given the central role of SUMOylation in regulating immune responses, pathogen survival, and intracellular signalling. Most potential therapeutic strategies can be adapted from cancer research, either through drug repurposing or by applying similar approaches to host–pathogen interactions. However, targeting SUMOylation or its regulatory interactors remains an underexplored therapeutic avenue in infectious disease contexts. As many pathogens exploit SUMO conjugation or deconjugation to modulate host defence systems, manipulating this pathway holds promise for restoring immune homeostasis and enhancing pathogen clearance (50).

One promising therapeutic direction involves enhancing host SUMOylation to reinforce cellular defences. SUMOylation of immune regulators such as IRF3, STAT1 and NF-κB inhibitors can augment antiviral signalling, stress responses and inflammation resolution. Strategies include the use of small molecules that stimulate SUMO E3 ligases or inhibit SUMO-specific proteases (SENPs), thereby maintaining SUMO conjugation during infection. For instance, increasing SUMOylation of transcription factors may enhance IFN-β production in viral infections like influenza and HIV, boosting antiviral gene expression (84).

In contrast, in situations where excessive or pathogen-induced SUMOylation promotes immune suppression, inhibiting SUMOylation becomes therapeutically valuable. Small molecule inhibitors targeting the E1 activating enzyme (SAE1/SAE2 complex) or the E2 conjugating enzyme (UBC9) have shown efficacy in reducing viral replication and immune evasion. For instance, SUMO pathway inhibitors impair HIV-1 integrase stability and reduce influenza NS1-mediated suppression of IRF3. In bacterial infections like tuberculosis, reducing Mtb-driven SUMOylation of transcriptional repressors restores macrophage activation and cytokine secretion (16).

Pathogen-specific SUMO–host interactions offer targeted therapeutic opportunities. Viral proteins like HSV-1 ICP0 and KSHV LANA that mimic SUMO E3 ligases or disrupt PML nuclear bodies can be selectively inhibited to restore antiviral structures (85). These targeted interventions promise reduced off-target effects compared to broad immunomodulation.

In fungal infections, modulating fungal SUMOylation can sensitize pathogens to oxidative stress or antifungal drugs, enhancing treatment outcomes. In tuberculosis and HIV co-infections, modulating SUMOylation could balance immune activation without inducing hyperinflammation, improving tolerance and efficacy of host-directed therapies (64).

Emerging technologies such as RNA interference (RNAi), CRISPR-based gene editing, and targeted protein degradation tools (e.g., PROTACs) are being explored to modulate SUMO-related genes with precision. These approaches offer potential for personalized medicine, allowing tailored interventions in patients with chronic or treatment-resistant infections (86).

While the therapeutic manipulation of SUMOylation is still in preclinical stages, its role in host–pathogen interactions makes it a compelling target. Continued research is needed to identify safe, specific modulators and to map SUMO-dependent pathways unique to different pathogens. Ultimately, integrating SUMO-targeted therapies into infectious disease management could transform how we approach intracellular infections and immune evasion.

While gene therapy remains an exploratory approach, studies demonstrating PIAS1 overexpression highlight the conceptual potential of modulating SUMOylation to restore host immune signalling in persistent infections. Experimental evidence indicates that enhanced PIAS1 expression improves cytokine production and phagosome maturation in infected macrophages, underscoring the promise—but also the experimental nature—of SUMO-based therapeutic modulation. In parallel, natural products and essential oils such as clove bud oil, anacardic acid, ginkgolic acid, and curcumin offer low-toxicity options for modulating SUMOylation indirectly. Anacardic acid and ginkgolic acid have proved their potential as SAE1 inhibitors in cancer and can be repurposed for modulating pathogen-induced SUMOylation (87, 88). These agents may work by altering redox signalling, transcription factor stability, or upstream inflammatory pathways that influence the SUMO machinery (89). Observations and literature survey suggest that essential oils, at sub-inhibitory concentrations, not only restore immune homeostasis but also enhance host defence signalling (90). Conversely, in viral infections where SUMOylation is hijacked to stabilize viral proteins, repress interferon responses, or promote latency, SUMO inhibition strategies are more appropriate. Small molecule inhibitors like ML-792 and TAK-981, initially developed for cancer, can disrupt viral manipulation of host SUMO machinery and restore antiviral responses (91). Additionally, blocking viral proteins that function as STUbLs could prevent the degradation of antiviral host factors like PML and Sp100 (92). These approaches may be particularly useful in persistent infections caused by HSV-1, KSHV, EBV, or HIV. Emerging RNA interference strategies could also be leveraged to transiently silence SUMO-conjugating enzymes (e.g., UBC9) or selectively knock down SUMO-modified host substrates exploited by pathogens. Looking ahead, future research should aim to refine the specificity and delivery of SUMO-modulating therapies by identifying pathogen-specific SUMO targets and SIM-containing effectors, mapping the crosstalk between SUMOylation and key immune pathways such as NF-κB, MAPKs, STATs, and autophagy (92), and developing high-throughput screening tools to identify natural and synthetic SUMO modulators. Engineering delivery platforms such as nanoparticles, exosomes, or viral vectors to enable tissue-specific and temporally controlled modulation of SUMO activity could enhance therapeutic precision (93). Moreover, integrating SUMO-based interventions with conventional antimicrobials may lead to synergistic effects, particularly in chronic or drug-resistant infections. As our understanding of SUMOylation deepens—especially its intersection with immune signalling, epigenetic regulation, and host defence networks—we envision SUMO-targeted therapies becoming a transformative addition to the arsenal against infectious diseases.

## Discussion

The host SUMOylation system plays a pivotal and multifaceted role in regulating immune responses, intracellular signalling, and transcriptional control during infection. Diverse pathogens—including bacteria, viruses, and fungi—have evolved to subvert or utilize SUMOylation to promote immune evasion, intracellular survival, and persistence. SUMOylation intersects with key signalling pathways such as NF-κB, JAK-STAT, and IRF, as well as epigenetic and vesicular trafficking mechanisms, demonstrating its broad regulatory influence (89).

A recurring theme among bacterial pathogens is their exploitation of SUMO-mediated regulation of vesicle trafficking and immune signalling. Mycobacterium tuberculosis, Salmonella enterica, and Legionella pneumophila manipulate SUMOylated Rab GTPases and SNARE components to remodel intracellular compartments and evade lysosomal degradation (94). These bacteria also target transcription factors and chromatin remodels to suppress cytokine production and antigen presentation, highlighting the dual spatial and transcriptional control exerted through SUMOylation (79). In viral systems, SUMOylation governs the stability, localization, and transcriptional effects of both host and viral proteins. HSV-1, HIV, influenza A virus, and KSHV have developed mechanisms to hijack SUMO machinery through SUMO-targeted degradation, altered immune regulation, or epigenetic modulation (13). In contrast, fungi rely primarily on their intrinsic SUMO machinery to regulate morphogenesis, stress adaptation, and virulence, indirectly contributing to immune evasion and persistence.

Crosstalk between SUMOylation and other PTMs such as ubiquitination, phosphorylation, and acetylation forms an integrated regulatory network that pathogens exploit to modulate host defences (50). Despite advances, critical knowledge gaps remain regarding temporal dynamics, cell-type specificity, and the identity of SUMO-modified substrates during infection (67).

Therapeutically, SUMOylation represents a promising yet complex target. Enhancing SUMO conjugation may strengthen immune responses in certain contexts, while inhibition may be required where pathogens induce hyper-SUMOylation to suppress immunity. Targeted modulation of pathogen-specific SUMO interactions offers opportunities for precision host-directed therapies (95). As emerging technologies such as CRISPR, RNAi, and chemical biology tools enable finer control of SUMO dynamics, integrating SUMO-targeted approaches with conventional antimicrobials could transform treatment paradigms for chronic and drug-resistant infections (96).
